# Design of next-generation ceramic fuel cells and real-time characterization with synchrotron X-ray diffraction computed tomography

**DOI:** 10.1038/s41467-019-09427-z

**Published:** 2019-04-02

**Authors:** Tao Li, Thomas M. M. Heenan, Mohamad F. Rabuni, Bo Wang, Nicholas M. Farandos, Geoff H. Kelsall, Dorota Matras, Chun Tan, Xuekun Lu, Simon D. M. Jacques, Dan J. L. Brett, Paul R. Shearing, Marco Di Michiel, Andrew M. Beale, Antonis Vamvakeros, Kang Li

**Affiliations:** 1 Barrer Center, Department of Chemical Engineering, Imperial College London, London, SW7 2AZ UK; 2 Electrochemical Innovation Lab, Department of Chemical Engineering, UCL, London, WC1E 7JE UK; 3 Department of Chemical Engineering, Faculty of Engineering, University of Malaya, 50603 Kuala Lumpur, Malaysia; 4 Department of Chemical Engineering, Imperial College London, London, SW7 2AZ UK; 5 Research Complex at Harwell, Rutherford Appleton Laboratory, Harwell Science and Innovation Campus, Harwell, Didcot, OX11 0FA UK; 6 School of Materials, University of Manchester, Manchester, Lancashire M13 9PL UK; 7 Finden Limited, Merchant House, 5 East St Helens Street, Abingdon, OX14 5EG UK; 8 ESRF – The European Synchrotron, 71 Avenue des Martyrs, 38000 Grenoble, France; 9 Department of Chemistry, University College London, 20 Gordon Street, London, WC1H 0AJ UK

## Abstract

Ceramic fuel cells offer a clean and efficient means of producing electricity through a variety of fuels. However, miniaturization of cell dimensions for portable device application remains a challenge, as volumetric power densities generated by readily-available planar/tubular ceramic cells are limited. Here, we demonstrate a concept of ‘micro-monolithic’ ceramic cell design. The mechanical robustness and structural integrity of this design is thoroughly investigated with real-time, synchrotron X-ray diffraction computed tomography, suggesting excellent thermal cycling stability. The successful miniaturization results in an exceptional power density of 1.27 W cm^−2^ at 800 °C, which is among the highest reported. This holistic design incorporates both mechanical integrity and electrochemical performance, leading to mechanical property enhancement and representing an important step toward commercial development of portable ceramic devices with high volumetric power (>10 W cm^−3^), fast thermal cycling and marked mechanical reliability.

## Abstract (web summary)

Miniaturized ceramic fuel cells are attractive for portable devices, but performance should be optimized. Here the authors report a micro-monolithic ceramic cell design for a tubular solid oxide fuel cell containing a multi-channel anode support with enhanced power density and stable operation.

## Introduction

Solid oxide fuel cells (SOFCs) convert chemical energy to electrical energy with high efficiencies >ca. 60%, and allow for wide fuel flexibility due to their operation at high temperatures (600–800 °C)^1,2^. For decades, advances in SOFC technology have focused on developing new materials, such as ceria-based ceramics for electrolyte and mixed ion-electron conducting (MIEC) perovskites cathodes^3–5^. More recently, protonic ceramic fuel cells (PCFCs), also known as proton-conducting SOFCs, have drawn increasing attention by demonstrating the capability of promising power output at an intermediate-temperature (IT) regime (400–600 °C), which is beneficial for the reduction of fuel cell cost^6–9^.

Despite these significant material advances, less progress has been made in terms of cell design with commercially available SOFC products being dominated by the conventional planar type design. However, the weak thermal cycling stability and the complex requirements for sealing still remain key challenges that prevent planar SOFCs from finding wide-spread adoption^10^. By contrast, high-temperature sealing is not an issue with tubular SOFCs; however, the area-specific power density is typically much lower than the planar design (cf. 0.2 W cm^−2^ vs 2 W cm^−2^, respectively)^10^. The advanced micro-tubular design provides significantly enhanced volumetric power densities through the miniaturization of cell dimensions to the millimetre scale, as well as superior thermal shock resistance, allowing rapid start-up/shut-down^11,12^. Despite these advances, cell mechanical integrity has always been an issue, as the long, high-aspect-ratio micro-tubes are extremely fragile against application of a longitudinal force, which results in practical difficulties for single-cell handling and stack assembly. Therefore, an ‘ideal’ design, which combines the features from both planar and tubular design types is desirable.

Thermal cycling stability, which is defined as the resistance to damage resulting from temperature changes, is one of the most important requirements for any type of SOFC technology as structural failure is one of the main reasons for high degradation rates^13,14^. In most cases, the SOFCs are examined post-mortem or indirectly with current/voltage monitoring, both of which offer limited information regarding the chemical, structural and mechanical evolution of the cell under operation. As such, there is a need for in situ non-destructive material characterization techniques that permit investigation of SOFCs in real time and provide direct insight into thermal cycling processes. Although absorption and phase contrast computed tomography (CT) techniques have been applied extensively to SOFC materials^15–18^, the study of full cells remains limited^19–21^. Moreover, XRD has been used extensively in the characterization of SOFC materials but spatially-resolved chemical tomography methods such as XRD-CT remain relatively emergent techniques. Recently, synchrotron X-ray diffraction computed tomography (XRD-CT) has proven to be a powerful materials characterization tool for non-destructive investigation of functional materials, like heterogeneous catalysts, catalytic membrane reactors and battery cells^22–24^, yielding spatially-resolved diffraction signals^25–37^. SOFC electrode materials have been investigated in the past with synchrotron X-ray diffraction but this is the first time, to the authors’ knowledge, that a full cell is investigated with XRD-CT^38–42^. Moreover, the non-destructive nature of synchrotron X-ray based techniques facilitates additional real-time examination of a complete cell.

Herein, we report a holistic approach to developing a new SOFC with both outstanding electrochemical performance and superior mechanical robustness, the ‘micro-monolithic SOFC’, which exhibits the structural features of a monolith, but is successfully miniaturized to the millimetre scale. This concept is based on ceramic honeycombs/monoliths that have high strength-to-weight ratios, as well as superior compression/shear strengths^43^. A custom-designed extrusion technique, assisted by a phase inversion process, has been employed to provide better flexibility in controlling and tailoring the morphology, as has been demonstrated previously^44–47^. This technique also offers a unique capability of decreasing geometrical dimensions considerably, which conventional fabrication techniques fail to achieve. The fabricated micro-monolithic SOFCs were characterized and compared with those with conventional micro-tubular geometry. In this work, XRD-CT has been employed for real-time examination of a complete cell during a thermal cycling test. Other properties associated with SOFC application, including mechanical strength, pore size distribution, gas permeability and electrochemical performances, were also investigated and discussed.

## Results

### Morphology characterizations

**Fig. 1:**
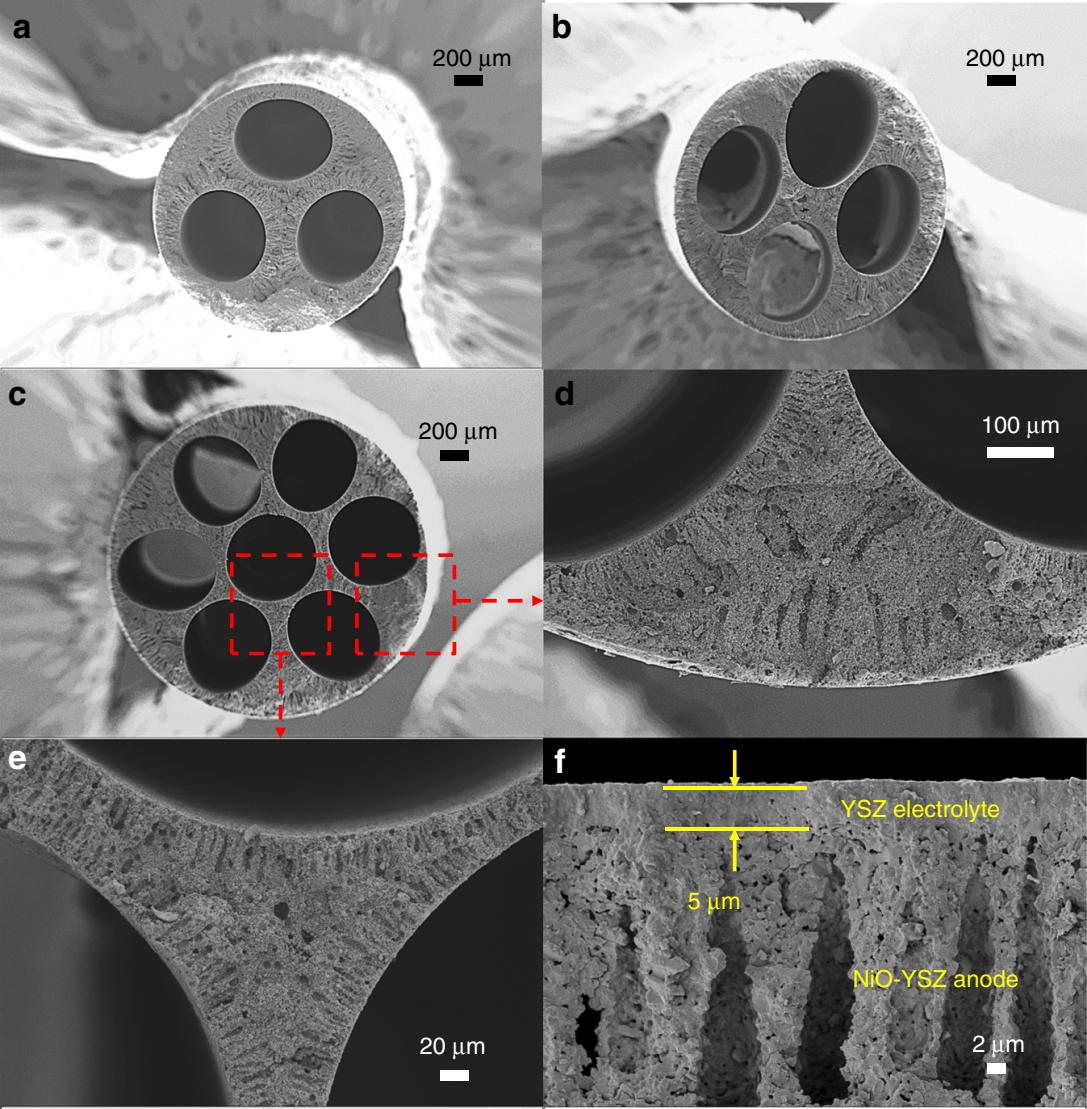
Various micro-monolithic anode supports after sintering at 1450 °C. SEM images of central-axis-view of: **a** 3, **b** 4, and **c** 7 channels; Cross-sectional images of **d** sponge between the shell and two channels in the 7-channel anode, **e** sponge between the central channel and surrounding two channels in the 7-channel anode; **f** close-up image of anode/electrolyte interface

Figure 1a–c show SEM images of sintered nickel oxide (NiO)-yttria-stabilized zirconia (YSZ) anode with different micro-monolithic geometries (fabrication details are in the Methods), with arrays of relatively uniform channels organized in the same configuration as the design of spinnerets used for phase inversion-assisted extrusion (as shown in Supplementary Fig. 1). The measured outer diameters of the sintered anode substrates are given in Supplementary Table 1. As can be seen, as the number of channels increased from 3 to 7 channels, there was a slight increase in the outer diameter from 2.1 to 2.5 mm, even though the spinnerets used have the identical outer diameter. This could have been due to the splitting of the internal coagulant which resulted in a more even distribution, and subsequently faster precipitation. Therefore, the elongation effect from the gravity of the nascent precursor has been inhibited. However, such outer diameters in the millimetre range are much smaller than that of any reported ceramic honeycomb/monoliths, which is also vital to increase the volumetric power densities of a stack.

Figure 1d and e present the cross-sectional image of a 7-channel anode as an example, as compared to the conventional (single) micro-tubular geometry (Supplementary Fig. 2a, b). The phase inversion process normally results in asymmetric structures including two typical morphologies, namely microchannels and sponge structures, which were observed in both micro-monolithic anodes and their conventional micro-tubular counterparts. A plurality of microchannels, which were evenly distributed around each channel, were introduced during the phase inversion process as a result of interfacial instability, and were well preserved during sintering, as proposed previously^48^.

The presence of such microchannels has been proved to effectively facilitate gas diffusion^49^, whereas the sponge region is the major contributor towards mechanical robustness and active sites. The dip-coated electrolyte had a thickness of approximately 5 µm (Fig. 1f) after co-sintering with the anode substrate. Measurements of sintering behaviour, reported in Supplementary Fig. 3, showed the maximum sintering rate of the YSZ electrolyte was approximately 2.5–3 times greater than that of NiO-YSZ, resulting subsequently in compressive stresses on the anode substrate. Hence, it was essential for the anode substrate to start sintering at temperatures ca. 200 °C lower than for YSZ to provide initial mechanical robustness, so preventing cracking or delamination.

After NiO was reduced to Ni using H_2_, the mean flow pore size of various micro-monolithic anode substrates was tested using a gas-liquid displacement (bubble point) porosimeter. Various micro-monolithic samples showed a similar mean flow pore size in the sponge region as their conventional single-channel counterparts, which is approximately 0.33 ± 0.02 µm (Supplementary Fig. 4). It is noteworthy that the micro-monolithic anode displayed a unique feature: the gradually varying thickness of the sponge region, offering short gas diffusion paths and decreasing mass transfer resistance of the fuel gas, as shown schematically in Supplementary Fig. 5. Therefore, even though the reduced anode substrates shared similar mean flow pore sizes with their conventional single-channel counterparts, the micro-monolithic anodes exhibited dramatically enhanced gas transport rates, as illustrated by nitrogen permeance results shown in Supplementary Fig. 6. This could enhance electrochemical performances significantly, as discussed in performance test section.

### Combined X-ray diffraction and absorption computed tomography

Synchrotron XRD-CT was employed to investigate the crystallographic properties within the fuel cell structure and therefore indirectly inspect the mechanical robustness/performance during thermal cycling. The 7-channel SOFC was selected for XRD-CT as the best-performing design among the various micro-monolithic geometries. Phase identification performed on the fresh SOFC XRD-CT data showed that the main crystalline phases present in the cell were NiO, YSZ and LSM (Supplementary Fig. 7b). After the reduction/activation, the NiO was reduced to metallic Ni and a thermal cycling test was performed, consisting of six 800 °C—room temperature (RT) cycles.

**Fig. 2:**
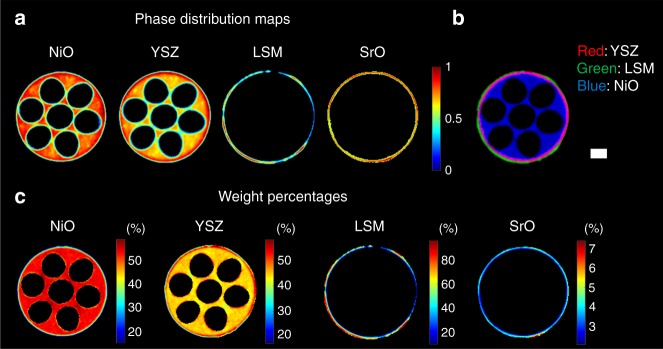
X-ray diffraction computed tomography of the fresh solid oxide fuel cell. **a** The phase distribution maps of NiO, YSZ, LSM and SrO as derived from the Rietveld analysis of the fresh SOFC XRD-CT datasets (colour bar indicates intensity in arbitrary units), **b** red green blue (RGB) image showing the distribution of YSZ (red), LSM (green) and NiO (blue), **c** weight % of all crystalline phases present in the SOFC. The scale bar corresponds to 0.5 mm

**Fig. 3:**
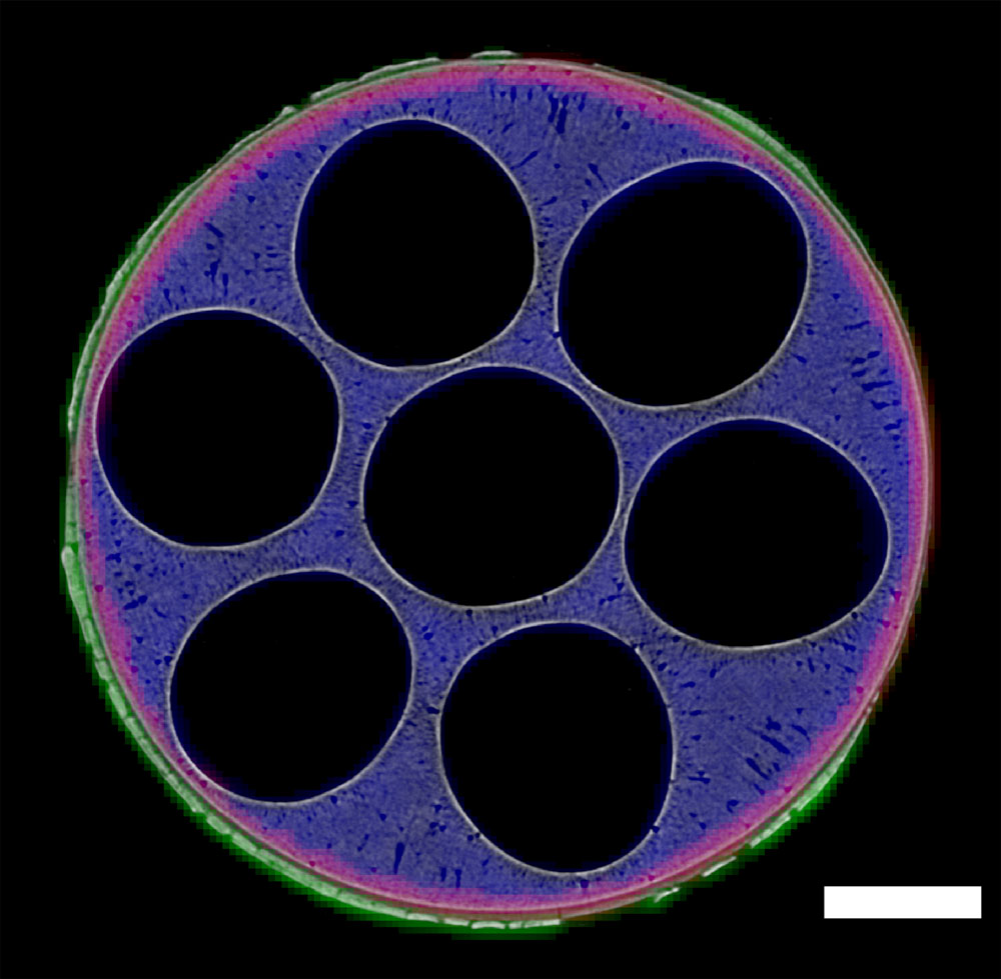
Micro-computed tomography and X-ray diffraction computed tomography images. XRD-CT maps of LSM (green), YSZ (red) and NiO (blue) have been overlaid on top of a micro-CT image collected at the same *z* position. The scale bar corresponds to 0.5 mm

Using the material-specific diffraction pattern peaks, we were able to map the three electrochemical components: anode, electrolyte and cathode through a full cross-section of the cell. This was achieved by performing Rietveld analysis of the XRD-CT data^26,28,30,36^. The results from the fresh SOFC are presented in Fig. 2 where the phase distribution maps of LSM, YSZ, NiO and SrO are shown (Fig. 2a). Additional 3D mapping of phase distribution can be seen in Supplementary Fig. 8 and Supplementary Video 1. These maps correspond to the scale factors for each phase. We were also able to create a mask based on the YSZ wt. % map and obtain a map of the electrolyte region (i.e., YSZ > 50 wt. %). This allowed us to create an RGB image (Fig. 2b) showing the three electrochemical components (red: electrolyte—YSZ, green: cathode—LSM, blue: anode—NiO). In Fig. 2c, the weight percentages of the various crystalline phases are also present showing that XRD-CT can also trace the minor SrO phase (<3 wt.%) which cannot be observed by conventional diffraction measurements. Such chemical contrast cannot be obtained with conventional micro-CT. Furthermore, in Fig. 3 the XRD-CT maps of LSM (green), YSZ (red) and NiO (blue) have been overlaid on top of a micro-CT image collected at the same *z* position.

**Fig. 4:**
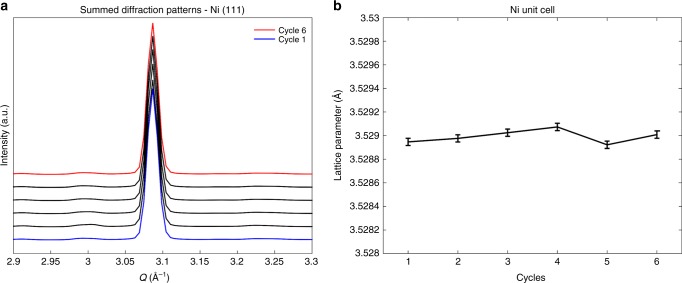
X-ray results from thermal cycling of the seven-channel solid oxide fuel cell. **a** The Ni (111) peak (highest intensity peak) with thermal cycling displaying minimal shift, **b** the Ni unit cell lattice parameter with thermal cycling

Presented in Fig. 4 is the highest intensity Ni diffraction peak (i.e., reflection (111)) of the summed XRD-CT diffraction patterns collected at room temperature, corresponding to the six thermal cycles (Q-values between 3.05 and 3.10 A^−1^, as shown in Fig. 2a). As depicted, there is insignificant change between the Ni peaks collected during the thermal cycling. However, to quantitatively confirm this, the lattice parameter a for the Ni unit cell was calculated. This was achieved by refining the Ni unit cell lattice parameter a during the Rietveld analysis of the XRD-CT data. As a result, the thermal stress could also be calculated for each thermal cycle (Supplementary Fig. 9), as described in a previous study^42^. From Fig. 4 it is clear the Ni lattice parameter a, thus thermal strain, changes by negligible amounts over the course of the six thermal cycles. Previously deviations in lattice parameters have been reported to change by up to 5 × 10^−3^ Å, however, this work reports deviations significantly less than 1 × 10^−3^ Å.

**Fig. 5:**
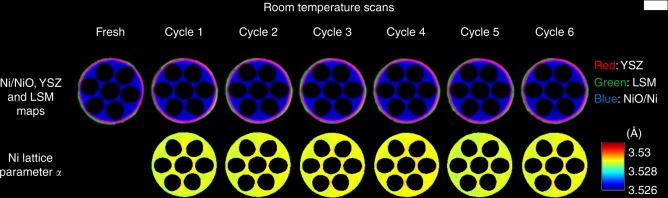
X-ray diffraction computed tomography of fuel cell during the thermal cycling. Row 1: Red green blue (RGB) images showing the distribution of the main SOFC components during the thermal cycling test (red: YSZ, green: LSM and blue: NiO/Ni). Row 2: the Ni unit cell lattice parameter a maps during the thermal cycling test as derived from the Rietveld analysis of the corresponding XRD-CT data. The scale bar corresponds to 0.5 mm

The crystallographic information presented in Fig. 4 and Supplementary Fig. 9 provide a quantitative assessment of the Ni lattice parameter a and strain during thermal cycling respectively but only in terms of bulk material i.e., localized weaknesses cannot be inspected. The spatially-resolved diffraction patterns obtained by XRD-CT allowed us to access spatially-resolved physico-chemical information about this real-life SOFC. The main results from the Rietveld analysis of the XRD-CT data during the thermal cycling test are presented in Fig. 5.

After the reduction-activation step, Ni is only present as metallic Ni (i.e., full reduction of NiO to Ni fcc has occurred). The LSM is also present for the duration of the experiment; the apparent changes in the LSM phase distribution map are not due to material migration but to sample vertical movement due to thermal expansion (slightly different positions in the sample are analysed). It can also be seen that the thickness of the pure electrolyte region does not change for the duration of the experiment indicating the superior stability of the cell during thermal cycling. Additionally, as with the summed diffraction data presented in Fig. 4, the Ni lattice parameter a and thermal strain could be extracted from the XRD-CT data. In Fig. 5, it can be seen that the Ni lattice parameter a is very homogenous throughout the cell (and corresponding thermal strain in Supplementary Figs 9 and 10) and remain consistent throughout the thermal cycling suggesting robust mechanical performance with thermal shock. This confirms the findings from the bulk information obtained from the summed diffraction patterns reported in Fig. 4.

**Fig. 6:**
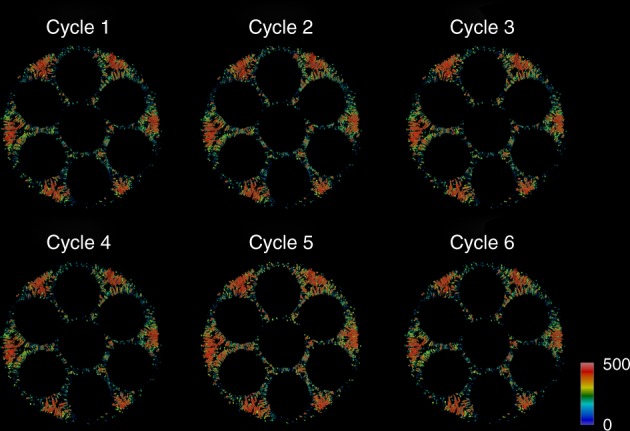
Volume rendering of pores during the thermal cycling of the solid oxide fuel cell. All micro-CT volume were aligned, then the pores were segmented and labelled according to their volume. The colour bar axes values have been chosen to enhance the contrast and make also the small pores in the microchannels visible (colour bar axes in arbitrary units)

Every XRD-CT scan was followed by a micro-CT scan at room temperature (Supplementary Fig. 11). Although these datasets do not offer chemical contrast, they can provide useful information regarding the morphology of the cell. In this work, the cathode LSM was first removed from the micro-CT data, then the pores of the cell were segmented and labelled according to their volume (Supplementary Figs 12 and 13). The volume rendering of these pores for each thermal cycle are presented in Fig. 6 where it can be clearly seen that the cell has retained its porous structure during the thermal cycling test (see also Supplementary Video 2). Specifically, both the small pores in the microchannels and the larger pores in the sponge regions (Supplementary Fig. 2) retain their shape and size for the duration of the thermal cycling test.

### Electrochemical performance

**Fig. 7:**
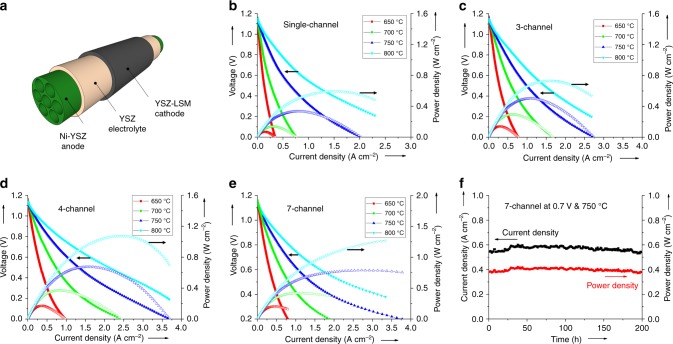
Electrochemical properties of micro-monolithic solid oxide fuel cells. **a** Schematic diagram of a 7-channel micro-monolithic SOFC; **b**–**e** Effect of current density (*j*) on cell potential difference (*U*) and power density in the temperature range 650–800 °C, with 30 mL min^−1^ H_2_ as the fuel and ambient air as oxidant; **f** terminal current density and power density at a voltage of 0.7 V at 750 °C for 7-channel cell under H_2_/ambient air for 200 h

A schematic diagram of a 7-channel micro-monolithic SOFC is shown in Fig. 7a, detailing the compositions of each component. Figure 7b–e report cell potential difference (*U*) – current density (*j*) – power density data for 1, 3, 4, and 7-channel cells operating with 30 mL min^−1^ of dry hydrogen fed to anode channels, and ambient air as oxidant at the cathodes. It is noteworthy that the active area was calculated based on the outer diameter of the cells, as shown in Supplementary Table 1. The measured open-circuit potential difference (OCPD) for all cells was 1.15–1.18 V at 650–800 °C, respectively, which is close to the theoretically expected values, indicating the 5 µm thick YSZ electrolyte was gas-tight. Maximum power densities for the single channel micro-tubular cell were 0.07, 0.14, 0.31 and 0.58 W cm^−2^ at 650, 700, 750 and 800 °C, respectively, whereas for 3-, 4- and 7-channel micro-monolithic cells, power densities at 800 °C were 0.67, 1.05 and 1.27 W cm^−2^, respectively, corresponding to enhancements of 16%, 81 and 120%, respectively. These increases are in good agreement with the nitrogen permeation results (Supplementary Fig. 5), which validated the effectiveness of micro-monolithic anodes in decreasing gas transport resistance. As discussed in the Morphology section, within this unique cross-sectional geometry that is highly asymmetric, the electrochemically active regions possess a thickness that is a mere 45 µm and distributed away from the mechanical support regions. Therefore, mass transport of the fuel has been significantly facilitated since there is no additional resistance from the support. Thus, the local hydrogen partial pressure remained almost unchanged compared to that in bulk, leading to enhanced performances. In addition, the power density of ca. 1.27 W cm^−2^ measured from the 7-channel cell is the highest ever achieved for micro-tubular design SOFCs with YSZ electrolytes. Excellent long-term stability of the design was demonstrated by dwelling the best-performing, 7-channel cell at 750 °C and 0.7 V. As shown in Fig. 7f, there is no observable degradation (<1%) in current density after 200 h of operation.

**Fig. 8:**
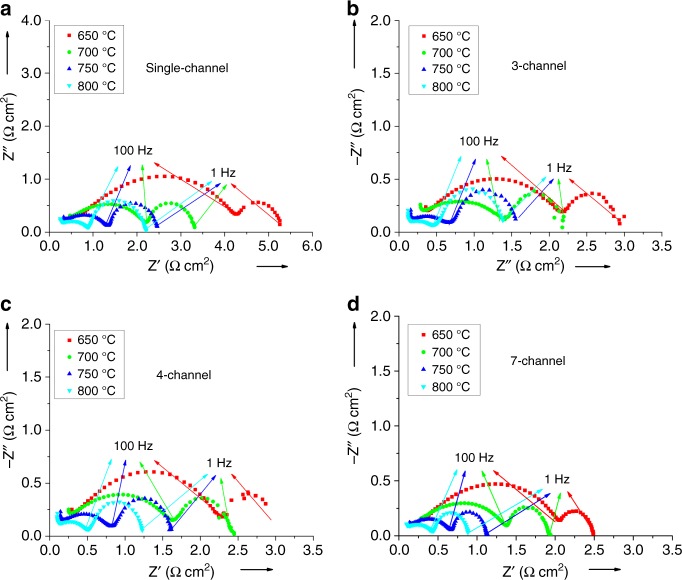
Electrochemical Impedance spectra. Effects of number of anode channels of micro-monolithic SOFCs and temperature on impedance spectra (10^−2^ to 10^5^ Hz) at open circuit potential

The influence of the number of anode channels on electrochemical performance can be explained further by electrochemical impedance spectra (EIS), as shown in Fig. 8. Each impedance spectrum comprised two major arcs; the low-frequency arc between 1–100 Hz represented a diffusion process. Whereas the shape of the low-frequency arcs was relatively independent of operating temperature, the high-frequency arc, due to activation polarization, decreased dramatically as the temperature was increased, in strong agreement with the literature^48^. In addition, the impedance, corresponding to activation polarization remained essentially the same for different micro-monolithic SOFCs, while the diffusion polarization was 1.1, 0.75 and 0.45 Ω cm^2^ for cells with 3, 4 and 7 anode channels, respectively. The diffusion polarization of the 7-channel cell was more than halved compared to that of the 3-channel cell, possibly due to differences in sponge-layer thickness, as illustrated in Fig. 1. Despite being non-uniform, the thinner regions in the 7-channel anode were 40–80 µm thick, compared with ca. 150 µm for the 3-channel anode support (cf. >200 µm for the single-channel cell, as shown in Supplementary Fig. 2a.

It is well understood that electro-active region extends ca. 10–20 µm from the electrolyte into porous anodes^50^, so most of the anodic volume is inactive, and functions as a mechanical support and current collector. As for the 7-channel anode, those thinner areas function as highly-active zone for electrochemical reactions with sufficient fuel inlet flow, while the geometry of the total cross-section provides excellent mechanical properties to the cell.

Notably, the miniaturization and introduction of monolithic structure in the anode support maintained the cell dimension within the ‘micro-tubular’ range, which was essential for a high volumetric power density. The maximum volumetric power density that micro-monolithic SOFCs could achieve was estimated, based on the closest triangle packing; the results are shown in Table 1. Compared to reported honeycomb-SOFCs with cross-sectional geometries of ca. 1 cm, the micro-monolithic SOFC with YSZ electrolyte showed significantly greater volumetric power densities, i.e., the 7-channel geometry had more than one order of magnitude performance enhancement (17 W cm^−3^). Moreover, all micro-monolithic SOFC geometries exhibited superior volumetric power densities compared to planar SOFC stacks, which are presently considered to deliver the greatest performance, with the 7-channel’s performances ca. twice that of planar equivalents. However, these lab-scale electrochemical performance results will not scale linearly with length. Nevertheless, micro-monolithic geometric design has great potential to be competitive with traditional planar design in terms of electrochemical performance.

**Table 1:** Comparison of volumetric power density with reported honeycomb-SOFCs

		T/°C	Volumetric power density/W cm^−3^	Ref.
This work	Single	800	9.6	N/A
	3-channel		10.4	
	4-channel		14.9	
	7-channel		17.0	
	Electrolyte			
Planar SOFC	N/A	N/A	3.0	^58^
	N/A	650	10.0	^59^
	YSZ	850	9.8	^60^
Honeycomb-SOFC	LSGM	800	0.50	^61^
	YSZ	850	0.29	^62^
	YSZ	850	0.35	^63^
	YSZ	900	0.78	^64^
	LSGM	800	0.60	^65^
	YSZ	1000	0.16	^66^
	YSZ	850	0.39	^67^

*LSGM* lanthanum strontium gallium magnesium oxide

### Mechanical properties

**Fig. 9:**
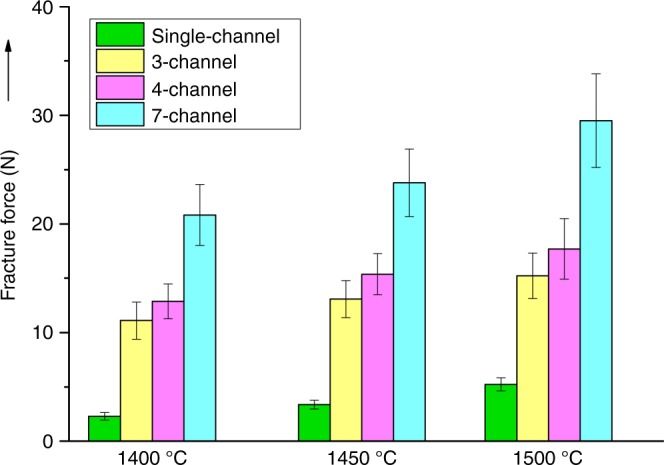
Mechanical properties. Effect of sintering temperature on fracture force of 1-, 3-, 4- and 7-channel anode samples. Miniaturized ceramic fuel cells are attractive for portable devices, but performance should be optimized. Here the authors report a micro-monolithic ceramic cell design for a tubular solid oxide fuel cell containing a multi-channel anode support with enhanced power density and stable operation

Generally, enhancement in gas transport may result in decreased mechanical strength, which is one of the essential properties required for the single-cell handling, stack assembling and long-term stability. Despite various sources of stress, such as axial compression, diametrical compression or bending, we believe that bending is more suitable to mimic the type of stress that stacks may experience from vibration in vehicular applications, as the high-aspect-ratio micro-tubes are extremely fragile against a longitudinally-directed force. Therefore, mechanical strength was characterized by the three-point bending method; results are shown in Fig. 9. Evidently, increasing sintering temperature increased bending loads prior to fracture for both micro-monolithic anode supports and the conventional single-channel designs. However, with increasing sintering temperature, porosities/gas permeation rates would decrease accordingly, requiring optimisation. Conversely, Fig. 9 showed micro-monolithic anodes sustain significantly greater fracture forces than conventional, single-channel micro-tubes; e.g. at 1450 °C, the bending load was enhanced from approximately 3–13 N for a 3-channel anode and 3–24 N for a 7-channel anode. After calculating the cross-section area and hence the bending strength of different samples, the values of ca. 210 ± 20 MPa for micro-monolithic anodes were found to be comparable with that for the single-channel geometry. This demonstrated the effectiveness of the micro-monolithic geometry in resisting the bending load, which is the most detrimental type of stress for high-aspect-ratio micro-tubes, without compromising the gas transport properties.

## Discussion

A holistic approach was developed for fabricating micro-monolithic anode supports that provide both structural integrity and high electrochemical performance. A phase inversion, extrusion-assisted process was used to fabricate solid oxide fuel cells with 3, 4, and 7 axial channels. Each was structurally and electrochemically characterized and compared to a conventional single-channel micro-tubular SOFC. The unique hierarchical pore structure, together with the microstructure from the phase inversion process, provided the micro-monolithic anode with superior gas transport, while the mean flow pore size remained comparable, which subsequently decreased concentration overpotential.

Peak power densities at 800 °C with H_2_ as the fuel were 0.67, 1.05 and 1.27 W cm^−2^ for 3, 4 and 7-channel cells, respectively, showing a performance increase of 16, 81, and 120% compared to the conventional micro-tubular SOFC (0.58 W cm^−2^). Good long-term stability was validated from the 200-h fuel cell operation of the 7-channel cell. Electrochemical impedance spectra revealed a significantly decreased concentration polarization loss compared to the single-channel geometry, which we believe is the reason for this increase. The micro-monolithic SOFCs also preserved micro-tubular design advantage of high volumetric power density; e.g., 17 W cm^−3^ for the 7-channel cell, which compares well with planar SOFC stacks and is more than one order of magnitude higher than that of the reported honeycomb-SOFCs.

Moreover, rather than compromising robustness, the micro-monolithic anode supports demonstrated superior bending fracture resistances than conventional designs, with bending loads increasing by 4–8 times as the number of channels increased. To the best of our knowledge this is the first time that a geometric design for ceramic fuel cells has demonstrated superior electrochemical performance while maintaining exceptional mechanical properties, and the application of such micro-monolithic design could well be extended to reversible solid oxide electrochemical cells for energy storage by cyclic fuel cell—electrolyzer operation, as well as recent intermediate-temperature proton ceramic fuel cells.

To better demonstrate the superior mechanical properties of this new micro-monolithic design, synchrotron XRD-CT has been applied to investigate the thermal cycling performance of a real-life SOFC during thermal cycling, illustrating excellent chemical/structural heterogeneity the micro-monolithic SOFC possessed during the cycling. This work not only demonstrates the feasibility to perform spatially-resolved studies of full cells, but also adds a very powerful characterization tool for the SOFC community to exploit and perform in situ and in operando chemical tomographic experiments.

## Methods

### Materials

Yttria-stabilized zirconia (8 mol% YSZ), nickel oxide (NiO) and lanthanum strontium manganite (LSM) were purchased from Inframat Advanced Materials and used as supplied. polyethersulfone (PESf) (Radal A300, Ameco Performance, USA), 30-dipolyhydroxystearate (Arlacel P135, Uniqema), and N-methyl-2-pyrrolidone (NMP, HPLC grade, VWR) were applied as the polymer binder, dispersant and solvent, respectively.

### Fabrication of micro-monolithic solid-oxide fuel cell

The micro-monolithic anode substrate was fabricated via a phase inversion-assisted process similar to that of single-channel hollow fibre, as described elsewhere^51^. Generally, a suspension composed of ceramic particles, solvent and polymer binder was homogenized for 4 days via planetary ball milling (SFM-1 Desk-top Miller, MTI Corporation, USA). Prior to being transferred into stainless steel syringes, the suspension was degassed under vacuum to fully eliminate trapped air bubbles. The whole spinning procedure is controlled by syringe pumps (Harvard PHD22/200 HPsi) to achieve precise control over extrusion rates of various components. It is noteworthy that the internal coagulant, de-ionized water, was split into required number of streams by a custom-designed nozzle before coming into contact with the suspension, to form the multi-channel structure. The precursor fibres were removed from the external coagulant bath after the phase inversion was completed, and subsequently dried and straightened at room temperature. Detailed designs of spinneret with adjustable nozzle numbers are depicted in Supplementary Fig. 1. Supplementary Table 2 illustrates the details of suspension compositions and spinning/sintering conditions. The ceramic mixture contained YSZ and NiO powder with a weight ratio of 40:60. The amount of polymer binder was 25 wt.% relative to the solvent. During the fabrication, a constant extrusion rate was maintained, but as the number of nozzles increases, a greater quantity of internal coagulant was required to fully open the channels. After the precursor fibre was dried and cut to the desired length, a high-temperature sintering process was undertaken at various temperatures for 6 h. Before some of the subsequent characterizations, the samples were reduced in pure hydrogen at 550 °C, converting NiO into Ni, which could better simulate the real operating conditions.

YSZ electrolyte was prepared by a dip-coating process. Anode precursors were dipped into ink and pulled up with a withdraw speed of 2 mm s^−1^. After the coated electrolyte was dried, the anode and electrolyte were co-sintered at 1450 °C in ambient air for 6 h. Finally, the dual-layer cathode was dip-coated onto the sintered half-cell that had been cut to a length of 5 cm. The length of the dip-coated cathode was controlled to be 1 cm. After both layers were dried, a sintering process at 1000 °C was undertaken for 1 h in air. The thickness of each cathode layer is approximately 15 µm.

### Experimental setup for synchrotron X-ray computed tomography measurements

The experimental setup used for the high-temperature treatment of the SOFC was similar to the one used previously^23^, as depicted in Supplementary Fig. 7. The SOFC investigated in this study was inserted into a quartz tube. The SOFC was glued on top of an alumina rod. The reactor cell (i.e., the SOFC and the alumina rod) was inserted inside a quartz glass tube (6 mm outer diameter, 5 mm inner diameter) and a four-way Swagelok piece was used to allow the use of two different gas streams. Air was used at the outer side of the SOFC (i.e., cathode side) and 2 % H_2_ in He at the inner side of the SOFC (i.e., anode side). The reactor cell was mounted into a gas delivery stub, itself mounted to a Huber goniometer head 1006 (to enable alignment). The goniometer was fixed to a rotation stage set upon a translation stage to facilitate the movements required for the CT measurement. An ID15A-developed furnace designed for CT experiments was used for the thermal cycling experiments. Temperature calibration was performed before the experiment using a thermocouple. Motorised stages were put in place to control the position of the furnace by the beamline computer.

### Synchrotron X-ray diffraction computed tomography measurements

XRD-CT measurements were made at the beamline station ID15A of the ESRF using a 90 keV monochromatic X-ray beam focused to a spot size of 40 μm × 20 μm (horizontal × vertical). 2D powder diffraction patterns were collected using the Dectris Pilatus3 X CdTe 2 M hybrid photon counting area detector. The total acquisition time per point was 20 ms and each XRD-CT scan lasted in total for 26 min. Initially, an XRD-CT scan was performed at the middle of the SOFC at ambient conditions. The XRD-CT scan consisted of 351 translation steps (translation step size of 20 μm) covering 0–180 ° angular range, in 176 steps. The detector calibration was performed using a CeO_2_ NIST standard. Every 2D diffraction image was converted to a 1D powder diffraction pattern after applying a 10% trimmed mean filter to remove outliers using the nDTomo^52^ and pyFAI software packages^53–55^. The data integration was performed with fast GPU processing. The final XRD-CT images (i.e., reconstructed data volume) were reconstructed using the filtered back projection algorithm.

### Synchrotron three dimensional X-ray diffraction computed tomography of fresh cell

A 3D-XRD-CT scan consisting of 21 XRD-CT datasets (each 40 microns apart) was also collected at the beamline station ID15A of the ESRF using a 90 keV monochromatic X-ray beam focused to have a spot size of 40 μm ×20 μm (horintal × vertical). Each XRD-CT scan was made with 161 translation steps (translation step size of 20 μm) covering 0–180 ° angular range, in 151 steps using the continuous rotation-translation data acquisition method^36,56^. The detector calibration and data treatment were performed in the same way as for the XRD-CT datasets collected during the thermal cycling experiment.

### Synchrotron micro-computed tomography measurements

The micro-CT measurements were performed at beamline ID15A of the ESRF using a 90 keV monochromatic X-ray beam. Radiographs were recorded with an X-ray imaging camera and the pixel size was 3.18 μm. The sample-to-detector distance was 2 m in order to enhance the contrast in the images (phase contrast). Each micro-CT scan consisted of 1500 projections (radiographs) covering an angular range of 0–180 ° (i.e., angular step size of 0.12 °). Flat-field and dark images were also collected prior to each micro-CT measurements and were used to normalize the acquired radiographs before the tomographic reconstruction. The tomographic data were reconstructed using the filtered back projection algorithm.

### X-ray diffraction computed tomography data treatment

TOPAS software was used for quantitative Rietveld refinement, on a voxel by voxel basis, with the reconstructed diffraction patterns^28,57^. Various figures presented in this work (e.g., phase distribution maps based on the scale factors or weight percentages, lattice parameters etc.) were created by importing the results from the refinements into MALTAB Unless stated otherwise, the Rietveld analysis of the XRD-CT data presented here was based on the intensity of the scale factors. Rietveld analysis was performed using the summed diffraction pattern of each XRD-CT dataset prior to the Rietveld analysis of the XRD-CT data in order to have a good starting model before performing the batch Rietveld analysis. The Ni thermal strain was calculated based on previously reported methods^42^, which includes calculating the change in Ni lattice parameter a with respect to the maximum value from all XRD-CT datasets. Topas v5 software was used to analyze the reconstructed diffraction data^57^.

### Thermal cycling experimental protocol

Initially, an XRD-CT scan was performed at the middle of the SOFC at ambient conditions. The temperature of the system was then increased to 800 °C with a ramp rate of 20 °C min^−1^ under the flow of air (cathode side, 100 mL min^−1^) and 20% H_2_ in He (anode side, 80 mL min^−1^). The concentration of the gases was kept constant for the duration of the thermal cycling experiment. The system was kept at 800 °C for 3 h to ensure the full reduction of the Ni anode. The furnace was then removed and the cell was exposed to ambient conditions. A room temperature XRD-CT and micro-CT scan were collected before the sample was reinserted into the furnace (i.e., by moving the furnace into the previous position) and completing the first thermal cycle. A waiting time of 5 min was applied before collecting any data. The total time spent with the sample exposed to ambient conditions was ca. 35 min (i.e., 5 min dwell time, 26 min for the XRD-CT scan and 4 min for the micro-CT scan and for the time needed to change the detector configuration from diffraction to absorption mode). The sample was kept at high-temperature for 35 min before removing the furnace and collecting the second set of CT scans. In total, 6 cycles were performed with the duration of the whole experiment being ca. 12 h.

### Phase identification and analysis of the X-ray diffraction computed tomography data

The phase identification of the fresh SOFC was performed using the summed diffraction pattern from the XRD-CT dataset collected at ambient conditions. The crystalline phases identified were: La_0.8_Sr_0.2_MnO_3_ (LSM) (ICSD: 51655), (ZrO_2_)_0.92_(Y_2_O_3_)_0.08_ (YSZ) (ICSD: 90889), SrO (ICSD: 163625) and NiO (ICSD: 9866). The results of the phase identification are presented in Supplementary Fig. 7b where a region of interest of the summed diffraction pattern is shown. These crystal structures were employed within the model used for the Rietveld analysis of the XRD-CT data. The phase identification of the activated/reduced SOFC showed that NiO reduced to metallic Ni (ICSD: 43397) and revealed the formation of the following new phases near/at the cathode electrode region: La_2_NiO_4_ (ICSD: 44121) and MnO (ICSD: 9864). This is attributed to the partial collapse of the LSM perovskite structure during the reduction process to the various metal oxides (i.e., MnO and SrO) and the reaction of the La species with Ni species to form a new phase, identified as La_2_NiO_4_. It should be noted though, that LSM remained present for the duration of the thermal cycling experiment without further decrease in quantity after the initial reduction.

Diffraction data were collected for CeO_2_ during the beamtime, and Rietveld analysis of the phase was used to create an instrument parameter file. The background was fit using a Chebyshev polynomial and the diffraction peaks were fit using a pseudo-Voigt profile function. A crystallographic information file (CIF) for CeO_2_ (ICSD: 72155) was obtained from the ICSD database and used for the Rietveld refinement. Rietveld analysis of the XRD-CT data was conducted using this instrument parameter file. The same data processing protocol was followed for all the XRD-CT data mentioned in this work. The results from the quantitative Rietveld refinement using the summed diffraction pattern of the XRD-CT data collected at room temperature of the fresh SOFC are shown in Supplementary Fig. 7c (Rwp = 5.853 % on 1710 observations).

### Characterizations

The morphology of the micro-monolithic geometry was observed using field emission scanning electron microscopy (FE-SEM) characterization (Gemini LEO 1525) under secondary electrons imaging (SEI) mode. Mechanical robustness was studied using a tensile tester (Instron Model 5543) with a load cell of 1 kN via a three-point bending method. Three sintering temperatures were investigated, namely 1400, 1450 and 1500 °C. Sintered anode samples were placed upon two sample holders with a span of 3 cm. 5 specimens were tested for each type of sample.

Mean flow pore size and gas transport property was investigated via N_2_ permeation tests at room temperature by gas-liquid displacement method using a bubble point porometer (PoroluxTM 1000). Reduced samples were sealed into the system using epoxy resin and the permeation flux of N_2_ was recorded with increased pressure difference. The gas permeance was subsequently calculated using Eq. 1:

1 $$P = \frac{Q}{{\pi LD_o \cdot {\mathrm{\Delta }}p}}$$

Here P denotes the permeability of N_2_ (mol m^−2^ s^−1^ Pa^−1^), L and Do represent length and the outer diameters of the fibre sample (m), respectively, and ∆*p* is the pressure difference across the micro-monolithic anode (Pa). The mean flow pore size was calculated using Laplace’s equation (Eq. 2):

2 $$r_p = \frac{{2\gamma }}{{{\mathrm{\Delta }}p}}cos\theta$$

Here *r*_*p*_ denotes the pore radius (m), *γ* represents interfacial tension at gas/liquid interface (N m^−1^)*, θ* is the contact angle.

Prior to the electrochemical performance test, silver wires of 0.2 mm diameter (99.99% purity, Advent Materials Ltd, UK) were wrapped along cathode and on exposed anode for current collection. Silver paste was painted to improve the contact between wires and electrode surface. A typical single cell of 5 cm was then sealed into alumina support tubes to construct a complete SOFC reactor. Wires from both electrodes were connected to a potentiostat/galvanostat (Iviumstat, Netherlands) for subsequent electrochemical performance tests. These measurements were conducted between 650–800 °C, with 30 mL min^−1^ of pure H_2_ fed to anode as the fuel and ambient air as oxidant. Electrochemical impedance spectroscopy (EIS) analysis was undertaken under open-circuit conditions in the frequency range of 10^5^−0.01 Hz with signal amplitude of 10 mV.

## Supplementary information

Supporting information

Supplementary information

Supplementary Video 1

Supplementary Video 2

## Data Availability

Copies of raw radially integrated XRD-CT data can be found at http://tiny.cc/NCOMMS18-33191A. Other data that support the findings of this study are available from the authors on reasonable request. See author contributions for specific datasets.
