# Supplementary material for: Design of next-generation ceramic fuel cells and real-time characterization with synchrotron X-ray diffraction computed tomography
Source: Nat Commun. 2019 Apr 2;10:1497. doi: 10.1038/s41467-019-09427-z (PMC6445146; doi:10.1038/s41467-019-09427-z)
Supplement: Supplementary file 1 — Supporting information [file 41467_2019_9427_MOESM1_ESM.docx]

**Supplementary Video 1. Phase distribution volumes of 3D-XRD-CT data.**

**Supplementary Video 2. Pore analysis of micro-CT data.**
