# Supplementary material for: Design of next-generation ceramic fuel cells and real-time characterization with synchrotron X-ray diffraction computed tomography
Source: Nat Commun. 2019 Apr 2;10:1497. doi: 10.1038/s41467-019-09427-z (PMC6445146; doi:10.1038/s41467-019-09427-z)
Supplement: Supplementary file 2 — Supplementary information [file 41467_2019_9427_MOESM2_ESM.pdf]

Supplementary Information

Next-generation Ceramic Fuel Cells: New Design and Real-life Characterization with Synchrotron X-ray Diffraction Computed Tomography

Tao Li[1,ł], Thomas M. M. Heenan[2,ł], Mohamad F. Rabuni[1,9], Bo Wang[1], Nicholas M. Farandos[3], Geoff H. Kelsall[3], Dorota Matras[4,5], Chun Tan[2], Xuekun Lu[2], Simon D. M. Jacques[6], Dan J. L. Brett[2], Paul R. Shearing[2], Marco Di Michiel[7], Andrew M. Beale[4,6,8], Antonis Vamvakeros[6,7]* and Kang Li[1]*

[1] Barrer Center, Department of Chemical Engineering, Imperial College London, London, SW7 2AZ, UK
[2] Electrochemical Innovation Lab, Department of Chemical Engineering, UCL, London, UK, WC1E 7JE
[3] Department of Chemical Engineering, Imperial College London, London SW7 2AZ, UK
[4] Research Complex at Harwell, Rutherford Appleton Laboratory, Harwell Science and Innovation Campus, Harwell, Didcot, OX11 0FA, UK
[5] School of Materials, University of Manchester, Manchester, Lancashire M13 9PL, UK
[6] Finden Limited, Merchant House, 5 East St Helens Street, Abingdon, OX14 5EG, UK.
[7] European Synchrotron Radiation Facility (ESRF), 71 Avenue des Martyrs, 38000 Grenoble, France
[8] Department of Chemistry, University College London, 20 Gordon Street, London WC1H 0AJ, UK.
[9] Department of Chemical Engineering, Faculty of Engineering, University of Malaya, 50603 Kuala Lumpur, Malaysia

ł The authors contributed equally to this work

Supplementary Figures

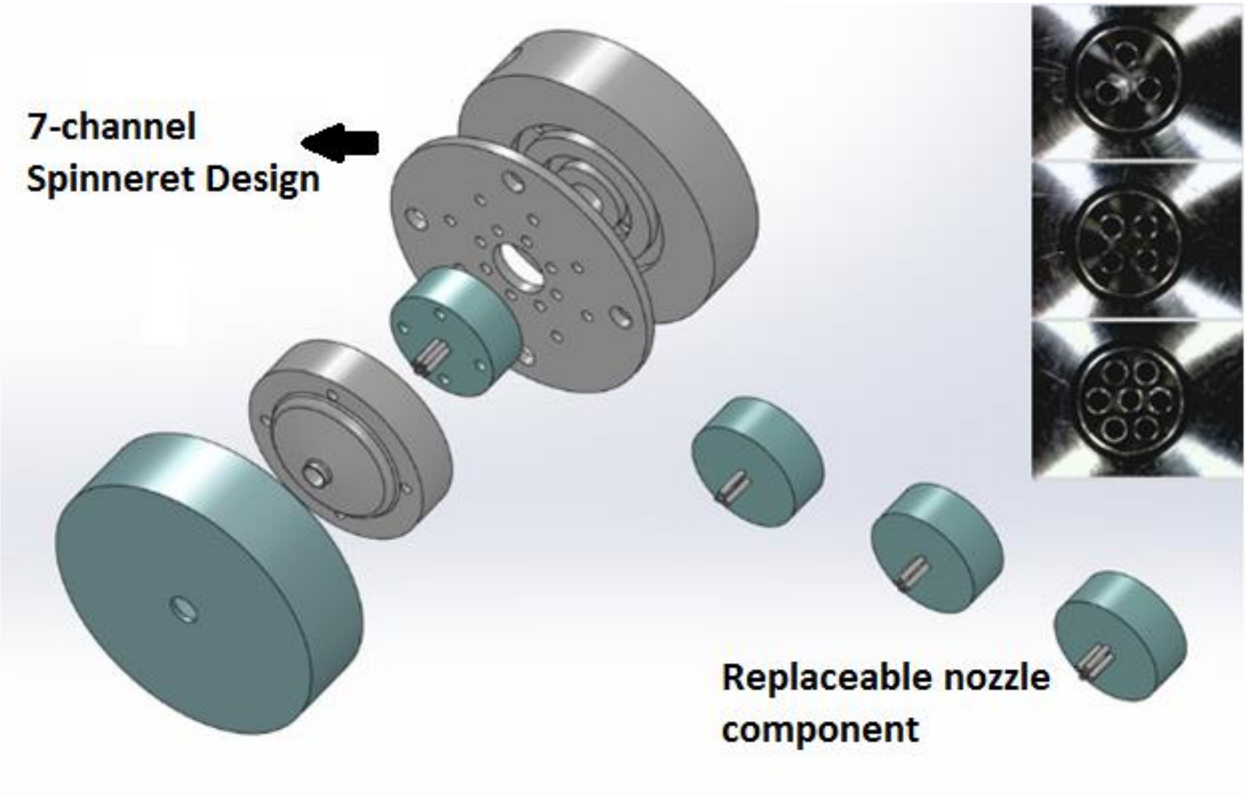

Supplementary Figure 1. Schematic diagram of the multi-nozzle spinneret.

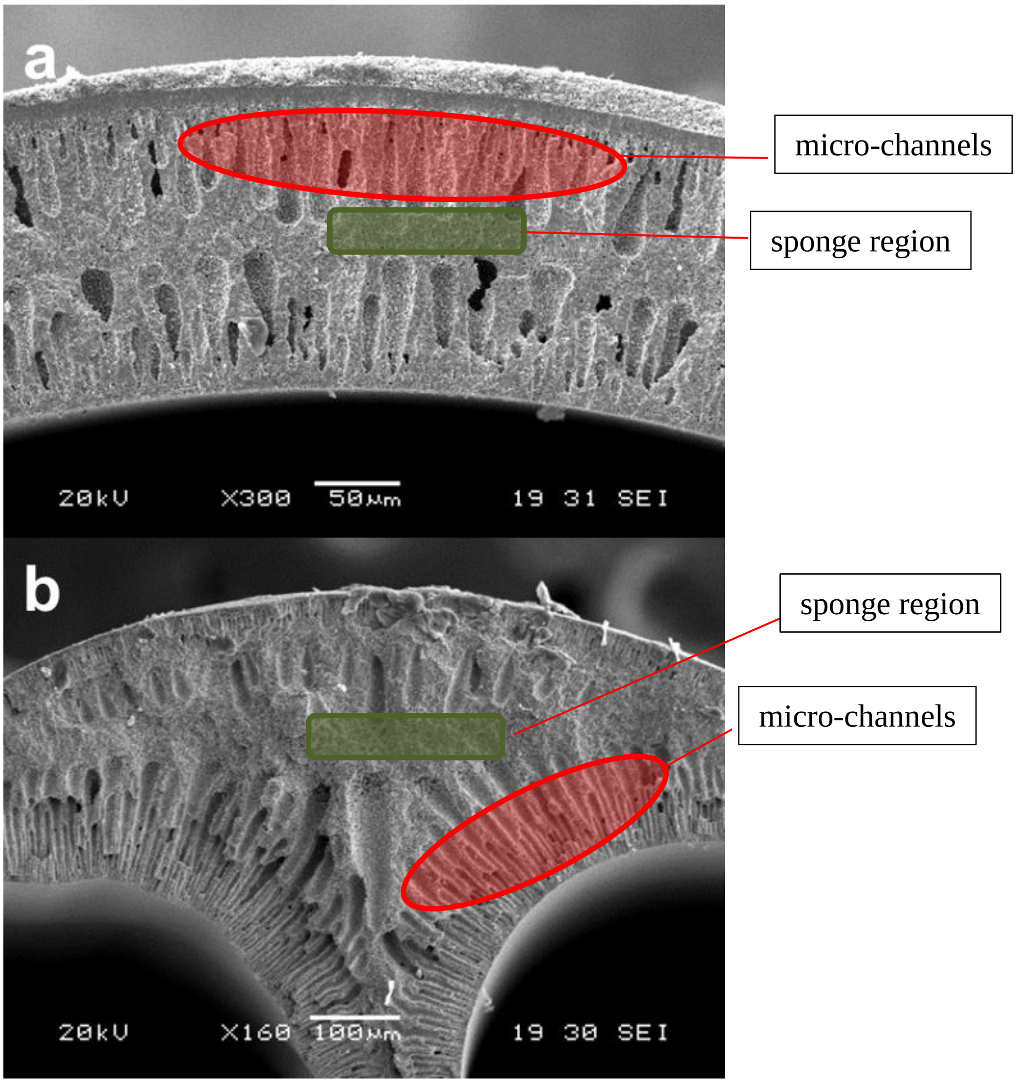

Supplementary Figure 2. **Close-up SEM images. a** cross section of single-channel hollow fiber; **b** cross section of sponge between the shell and two channels in the 7-channel anode.

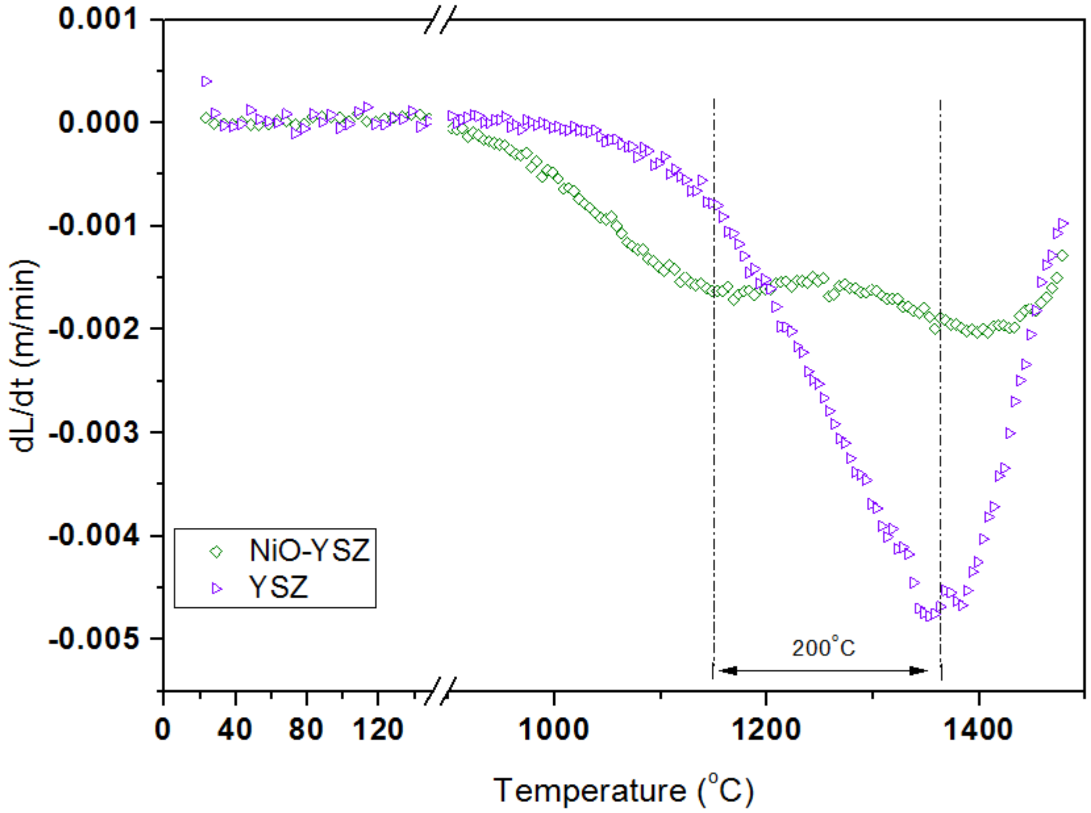

Supplementary Figure 3. Dilatometer study of anode and electrolyte materials. Sintering rate data for NiO-YSZ anode and YSZ electrolyte materials.

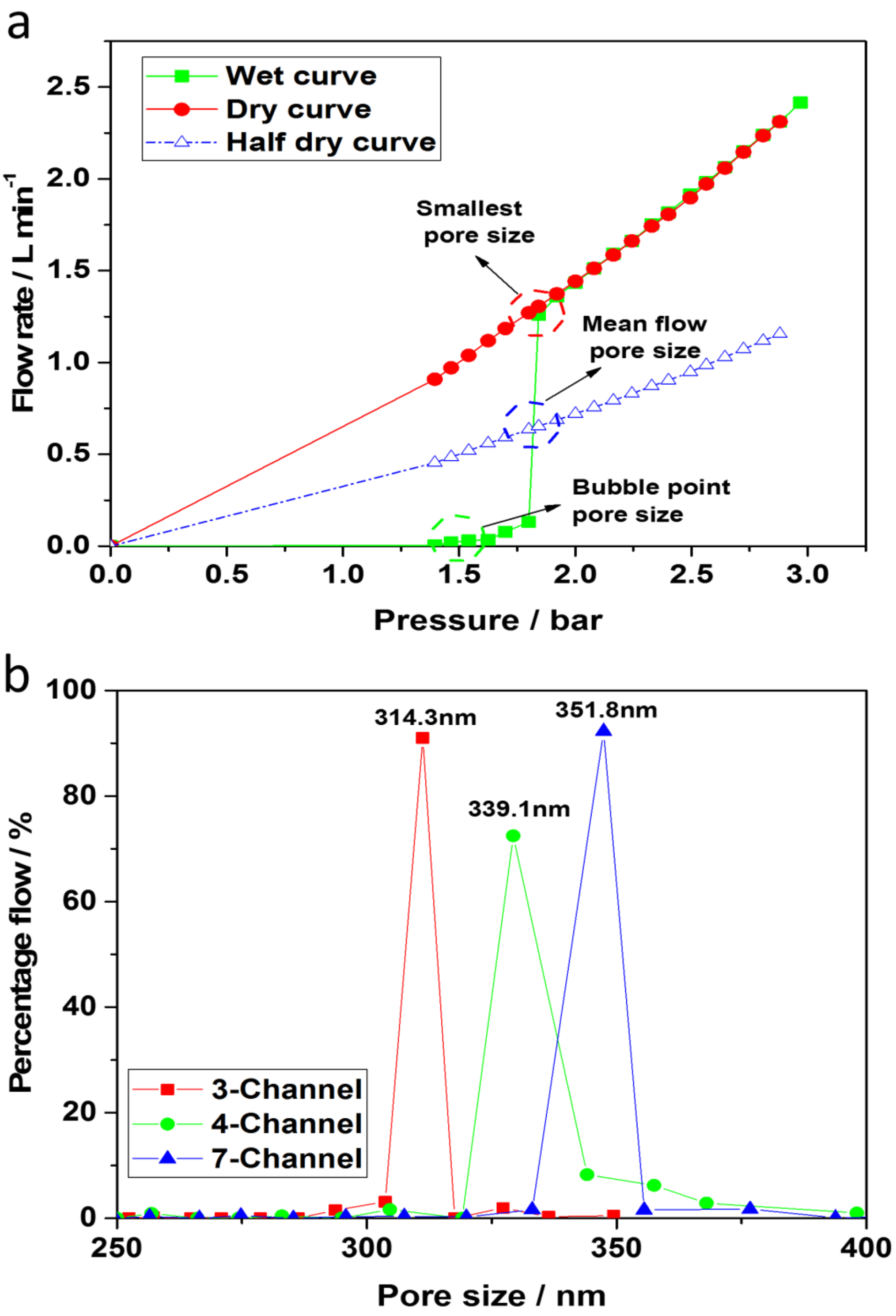

Supplementary Figure 4. Bubble point porosimeter curves. a wet, dry and half-dry curves for 7-channel sample; **b** pore size distribution of 3, 4 and 7-channel samples.

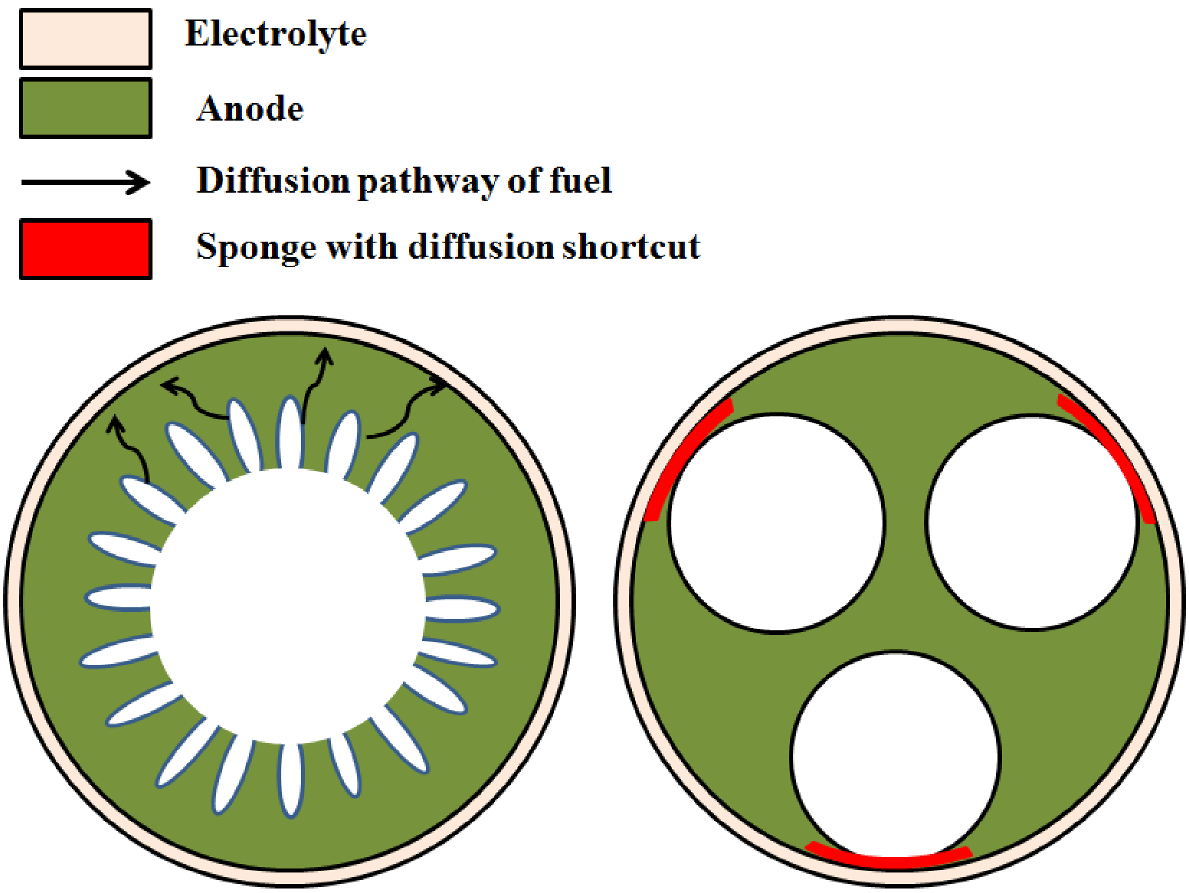

Supplementary Figure 5. Schematic diagram of the gas diffusion pathway. (left) traditional micro-tubular anode; (right) 3-channel anode

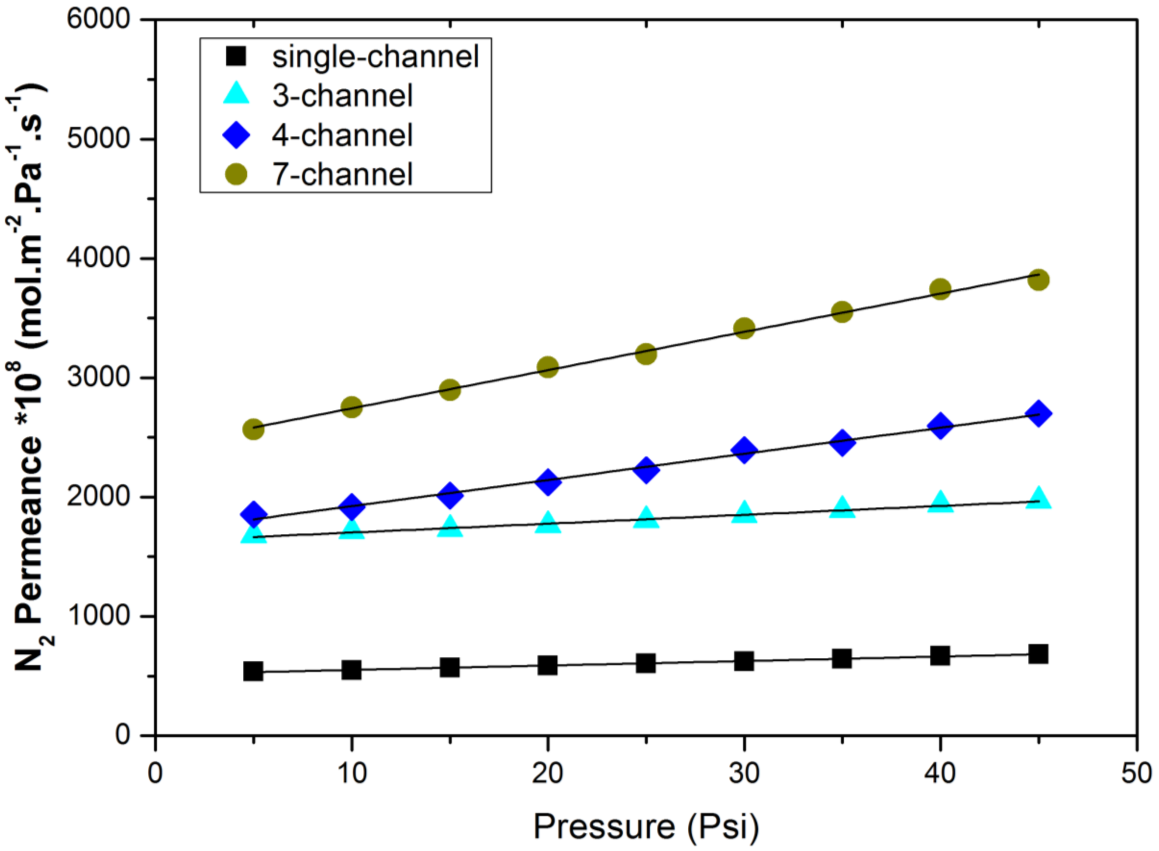

Supplementary Figure 6. The N_2 permeation test. N_2 permeance as a function of pressure difference of 3, 4, and 7-channel and conventional single channel (reduced) anodes at room temperature.

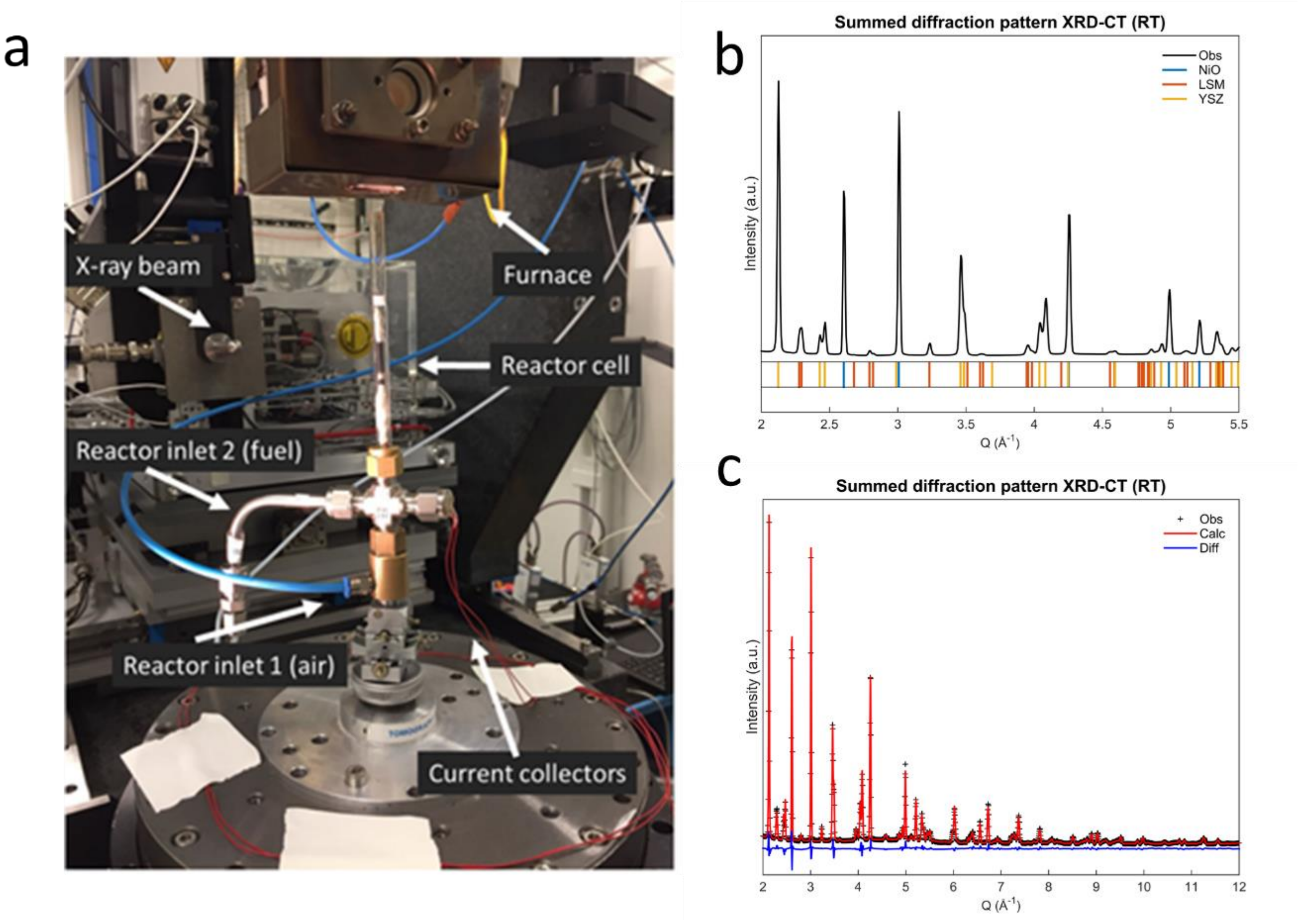

Supplementary Figure 7: Experimental information for the synchrotron x-ray diffraction computed tomography: **a** Photograph of the experimental setup used for the in situ XRD-CT measurements at the ID15A beamline of the ESRF, **b** Phase identification of the fresh SOFC. Black line: the summed diffraction pattern from the room temperature XRD-CT scan, Blue ticks: NiO, Orange ticks: LSM, Yellow ticks: YSZ, **c** Quantitative Rietveld refinement using the summed diffraction pattern from the room temperature XRD-CT dataset of the fresh SOFC.

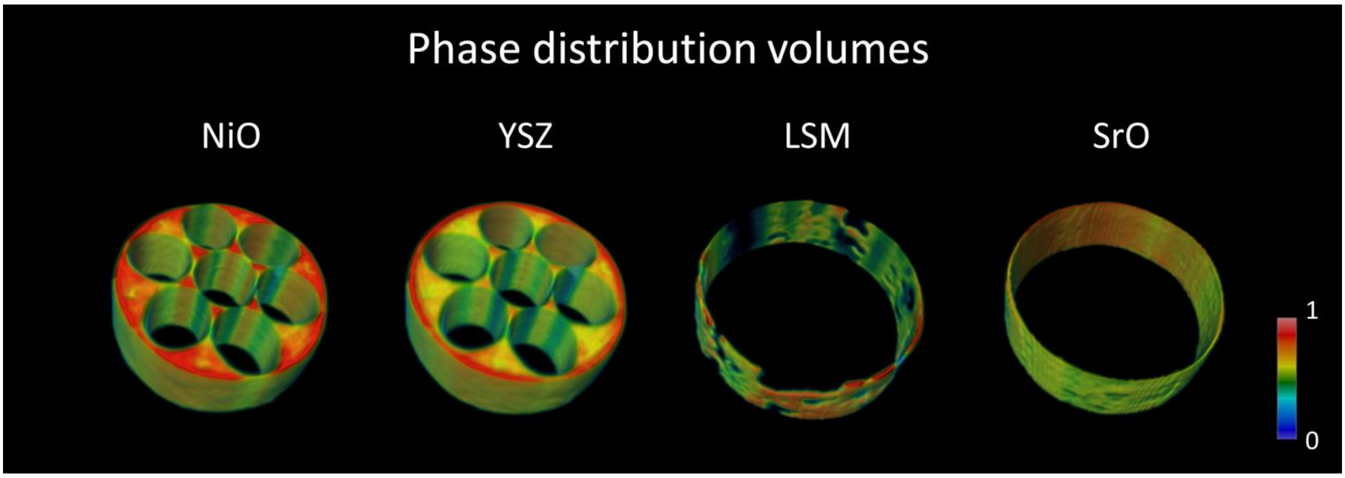

Supplementary Figure 8: 3D-XRD-CT of a fresh SOFC. The volumes correspond to the scale factors of the NiO, YSZ, LSM and SrO phases as derived from the Rietveld analysis of the XRD-CT data.

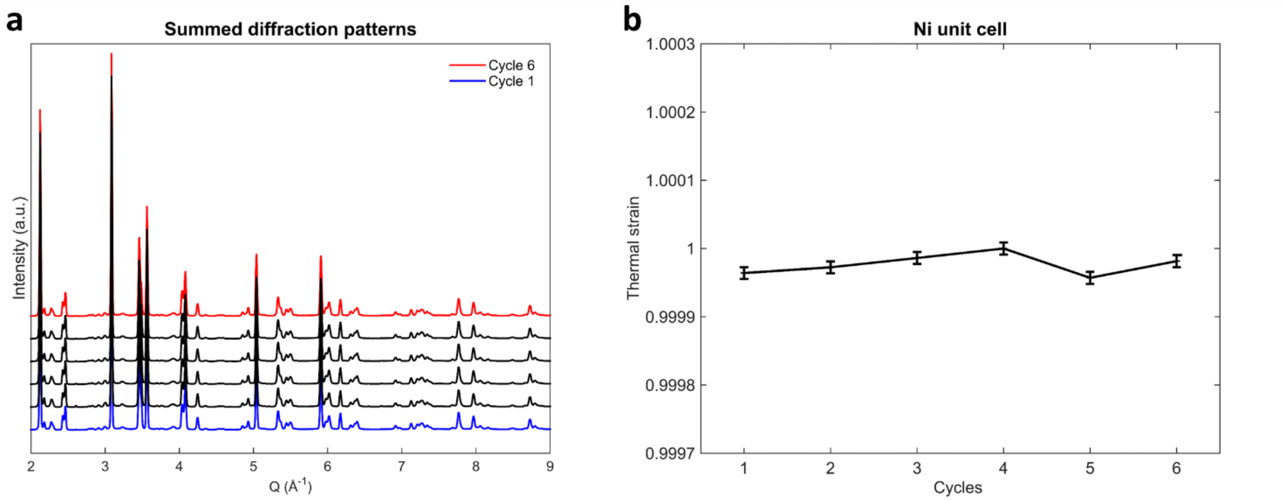

Supplementary Figure 9: Summed diffraction patterns and Ni thermal strain. Left: The summed diffraction patterns from the six XRD-CT datasets obtained during the thermal cycling experiment. Right: The calculated Ni thermal strain during the thermal cycling experiment including error bars.

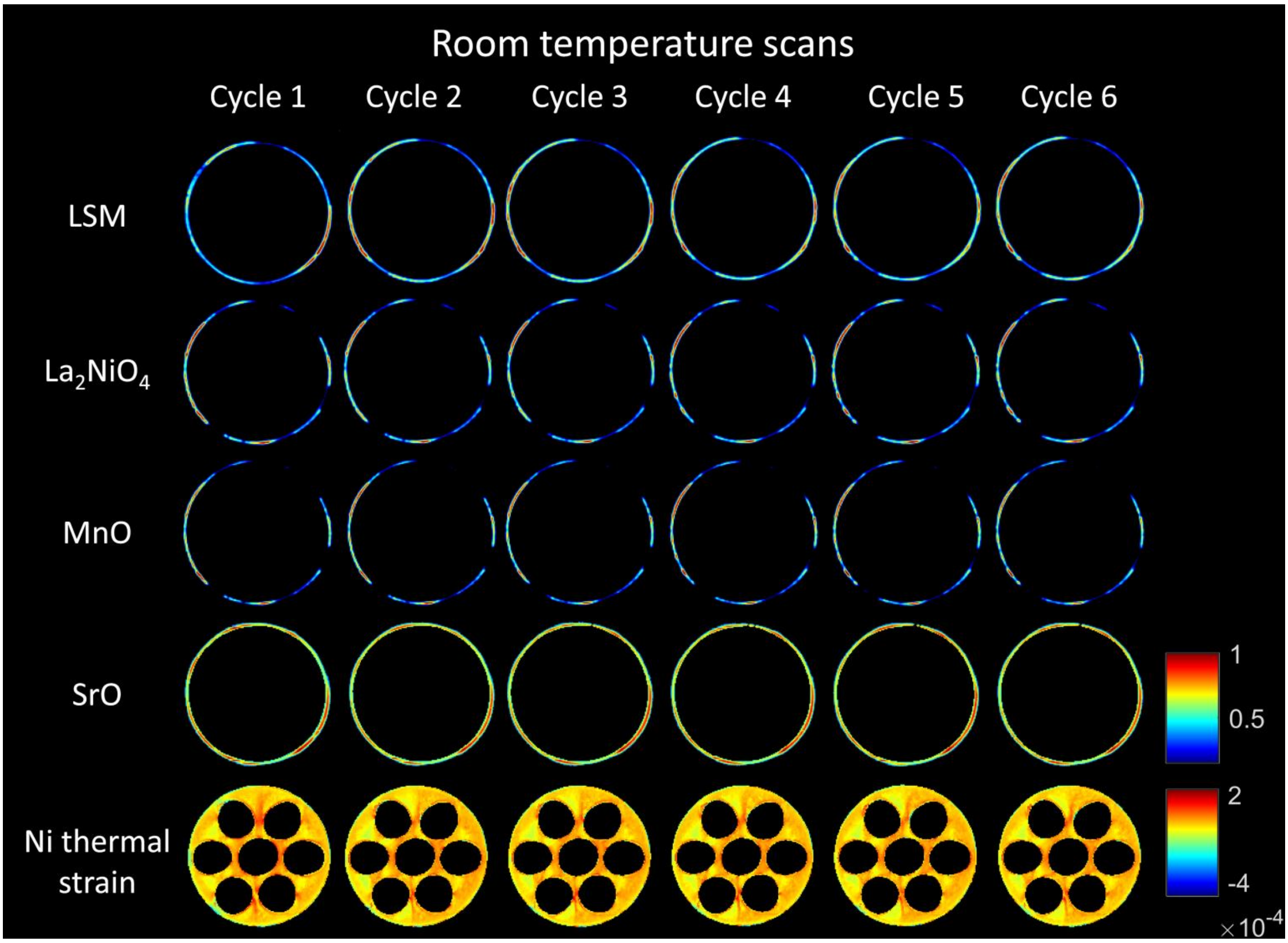

Supplementary Figure 10: SOFC behaviour during the thermal cycling. Rows 1-4: Phase distribution maps of LSM, La_2NiO_4, MnO and SrO as derived from the Rietveld analysis of the XRD-CT data collected during the thermal cycling experiment. Row 5: Map corresponding to Ni thermal strain (color bar indicates intensity in arbitrary units).

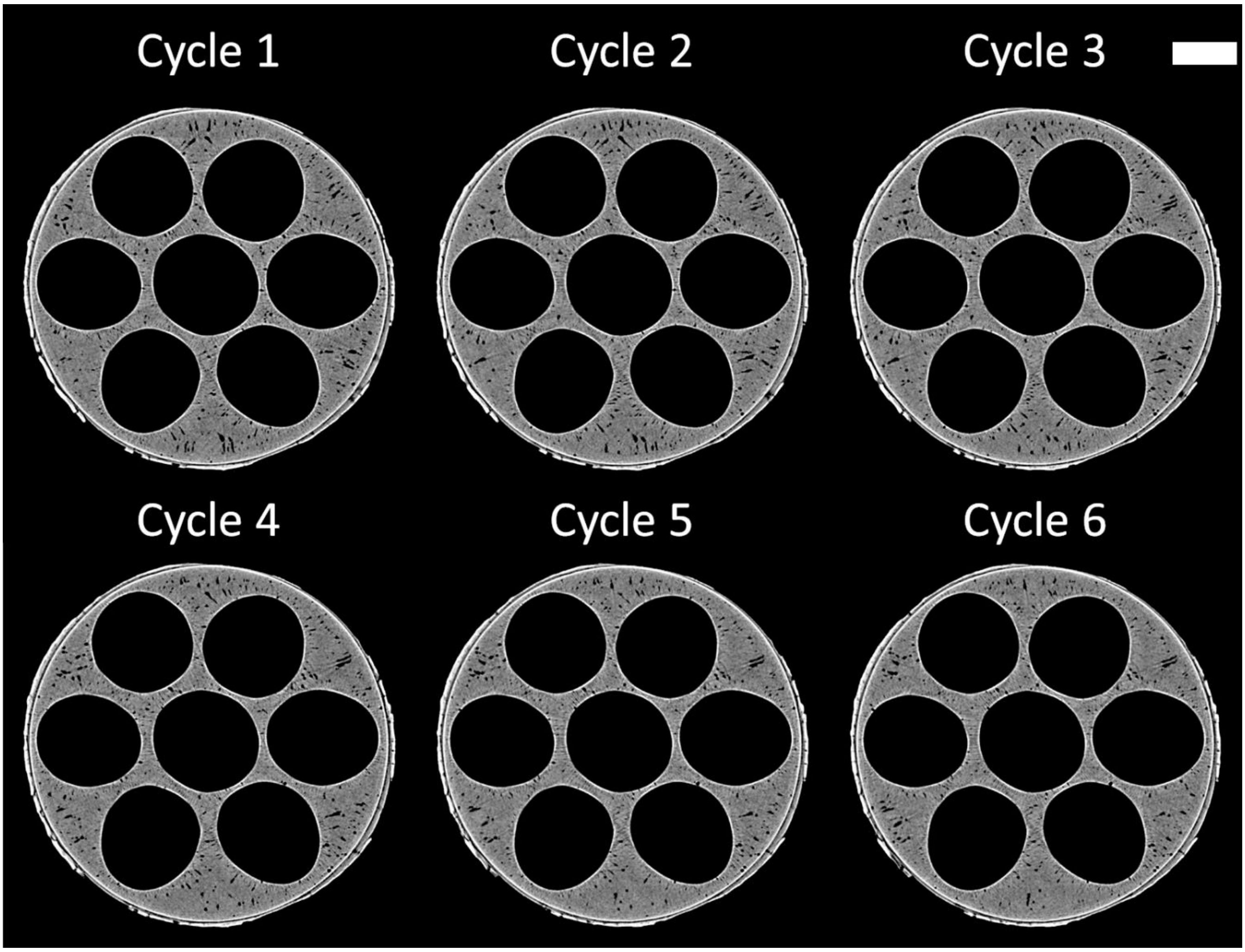

Supplementary Figure 11: Micro phase-contrast CT images collected at the same position as the XRD-CT data. It can be seen that the porous structure of the cell is maintained during the thermal cycling and no delamination is observed. The scale bar corresponds to 0.5 mm.

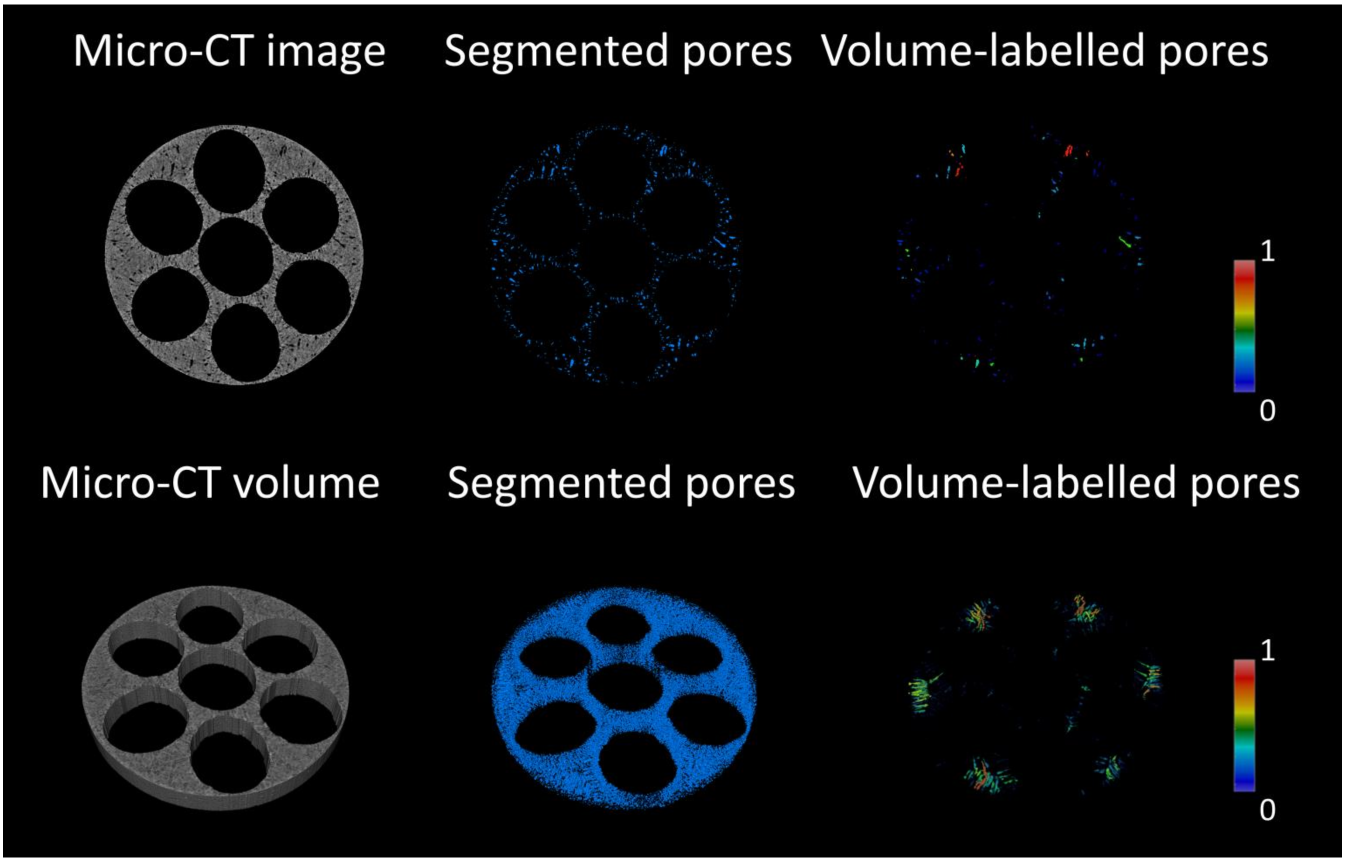

Supplementary Figure 12: Creating to volume rendeting of the SOFC pores. First the LSM cathode was removed from each micro-CT volume. The pores were then segmented using in-house MATLAB scripts and labeled according to their volume using the MorphoLibJ plugin of the ImageJ software[10]. Row 1: The process for each micro-CT image. Row 2: Volume rendering of the micro-CT data, segmented pores and labelled pores respectively. The colourbar axes values have been normalised which shows that the pores in the sponge region of the SOFC are significantly larger than the pores in the micro-channels (colour bar axes in arbitrary units).

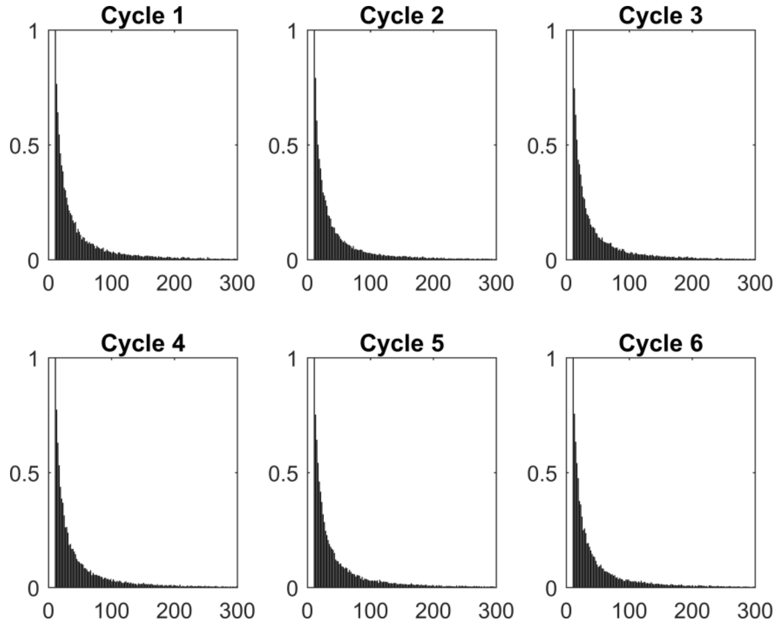

Supplementary Figure 13: Histograms of the labeled SOFC pores. It is apparent that the pore size distribution is not changing during the thermal cycling. Any minor changes should be attributed to artefacts during the segmentation process which introduces errors.

Supplementary Tables

Supplementary Table 1. Dimensions of single-channel and micro-monolithic anode.

	OD / mm	Channel diameter / mm	Active area / cm^2
Single-channel	1.96±0.02	1.65±0.01	0.615
3-channel	2.10 ±0.05	0.67±0.03	0.665
4-channel	2.30±0.04	0.70±0.02	0.728
7-channel	2.50±0.05	0.75±0.03	0.792

Supplementary Table 2. Suspension compositions and fabricating conditions of single and micro-honeycomb anode substrates

		Single-channel	3-channel	4-channel	7-channel
Suspension compositions	Ceramic (wt.%)	66.3 (NiO:YSZ=3:2)			
	Solvent (wt.%)	26.5			
	PESf (wt.%)	6.6			
	Additive (wt.%)	0.5			
Fabrication	Extrusion rate (ml/min)	8	8	8	8
	Bore liquid rate (ml/min)	6	7	8	10
	Air gap (cm)	1			
Sintering	Temperature (°C)	1400, 1450, 1500			
	Dwelling time (h)	6			

Supplementary References

10 Legland, D., Arganda-Carreras, I. & Andrey, P. MorphoLibJ: integrated library and plugins for mathematical morphology with ImageJ. *Bioinformatics* **32**, 3532-3534, doi:10.1093/bioinformatics/btw413 (2016).
